# IL-6 and IL-10 in the serum and exfoliated cervical cells of patients infected with high-risk human papillomavirus

**DOI:** 10.1371/journal.pone.0248639

**Published:** 2021-03-22

**Authors:** Camila Mareti Bonin-Jacob, Larissa Zatorre Almeida-Lugo, Marco Antonio Moreira Puga, Ana Paula Machado, Cacilda Tezelli Junqueira Padovani, Mariana Calarge Noceti, Alda Maria Teixeira Ferreira, Carlos Eurico dos Santos Fernandes, Júlio César Possati Resende, Adriane Cristina Bovo, Inês Aparecida Tozetti

**Affiliations:** 1 Postgraduate Program of Infectious and Parasitary Diseases from Medicine School, Federal University of Mato Grosso do Sul (UFMS), Campo Grande MS, Brazil; 2 Bioscience Institute, UFMS, Campo Grande, MS, Brazil; 3 Multicentric Program of Post-Graduation in Biochemistry and Molecular Biology of the Institute of Biosciences, UFMS, Campo Grande, MS, Brazil; 4 Department of Cancer Prevention, Barretos Cancer Hospital, Barretos, São Paulo, Brazil; 5 Department of Cancer Prevention, Barretos Cancer Hospital, Campo Grande, MS, Brazil; Rudjer Boskovic Institute, CROATIA

## Abstract

Persistent infection by high-risk human papillomavirus (HR-HPV) is the main cause of cervical cancer and its precursor lesions. While some cytokines help immune cells in virus clearance, others contribute to the persistence of infection and neoplastic progression. Here, the levels of interferon (IFN)-γ, tumor necrosis factor (TNF)-α, interleukin (IL)-10, IL-6, IL-4, and IL-2 were quantified in the serum and exfoliated cervical cells (ECCs) of patients with HR-HPV, and the presence of IL-6^+^ cells was investigated in uterine cervix biopsies. Cytokine levels in the serum and ECCs of 26 HR-HPV DNA-positive patients and 18 HPV DNA-negative patients were measured using flow cytometry. Fifteen uterine cervix biopsy samples embedded in paraffin were subjected to immunohistochemical analysis for the detection of IL-6^+^ cells. HR-HPV-positive patients showed increased IL-6 and IL-10 in the ECCs and serum, respectively. Compared with HPV DNA-positive patients, HPV DNA-negative patients had higher levels of IL-6 in ECCs. Patients with multiple infections of HPV had higher levels of IL-6 in their ECCs than those with a single infection. Immunostaining of uterine cervix biopsy samples revealed no differences in IL-6 expression between the different classes of histopathological lesions. However, differences were observed in the expression levels of IL-6 and IL-10 at the systemic and local levels in HR-HPV-positive patients without cervical lesions. Considering the functional characteristics of these cytokines, it can be inferred that such patients are prone to persistent HPV infection.

## Introduction

Cervical cancer is the fourth most common cancer among women worldwide [1]. Persistent infection with high-risk human papillomavirus (HR-HPV), especially HPV16 and HPV18, is mainly responsible for the development of cervical cancer and its precursor lesions, including squamous intraepithelial lesion (SIL) [2, 3]. HPV infection in the female genital tract has a transient pattern, and most immunocompetent individuals (70–90%) can eliminate the virus within 12 to 24 months of diagnosis [4, 5]. However, about 10% of patients fail to show viral clearance and remain infected [3].

Viral clearance and control of the progression of virus-induced lesions are mediated through an active immune response in immunocompetent individuals. In particular, the cell-mediated immune response plays an important role in the control of both HPV and cervical lesions [6, 7]. During this process, the cytokines secreted by both T cells and accessory cells such as keratinocytes are extremely important for the regulation and control of cellular immunity. Some cytokines help the cellular immune response eliminate and control HPV infection, including interferon (IFN)-γ, which enhances the expression of the major histocompatibility complex (MHC) in tumor cells and inhibits angiogenesis [8] and tumor necrosis factor (TNF)-α, which exhibits antitumor properties and inhibits the growth of some cell lineages transformed by HPV [9]. Other cytokines, such as interleukin (IL)-6, IL-10, and IL-17, have been associated with the persistence of HPV infection and the occurrence of favorable conditions to facilitate the development of neoplastic lesions [10–13]. Moreover, the autocrine secretion of IL-4 promotes increased survival and resistance to cell death in CD133^+^ cervical cancer stem cells (CSC) [14].

Because HPV infection is restricted to the epithelium and both local and systemic immunity may be related to virus clearance or persistence of HPV infection, the levels of cytokines IFN-γ, TNF-α, IL-10, IL-6, IL-4, and IL-2 were measured in the exfoliated cervical cells (ECCs) and serum of patients positive for HR-HPV DNA and negative for HPV DNA, a majority of which do not have cervical lesions. The expression of IL-6 in cervical stromal cells was also evaluated to better understand the behavior of these cytokines during HR-HPV infection.

## Materials and methods

### Study population

The samples used in this study were obtained from a previous study in which 410 women older than 18 years were subjected to identification and genotyping of HPV as well as the identification of the presence of co-infections [15]. The case group was composed of serum and exfoliated cervical cell (ECC) samples from 26 of 410 women positive for HR-HPV DNA selected using non-probability sampling for convenience. The control group was composed of serum and exfoliated cervical cell (ECC) samples from 18 women negative for HPV, randomly selected from the group of women negative for HPV (n = 373/410). HPV genotype assays were performed via polymerase chain reaction using PGMY primers, followed by type-specific PCR (TS-PCR) and restriction fragment length polymorphism (RFLP) S1 and S2 Tables. Samples that were not genotyped through TS-PCR and RFLP were sequenced using an ABI-Prism 3500 Genetic Analyzer (Applied Biosystems, Foster City, CA, USA) [15].

None of the patients included in the present study had undergone a hysterectomy or had exposure to immunosuppressive drugs, and none were pregnant or in their menstrual period. These patients were also negative for *Chlamydia trachomatis* and *Trichomonas vaginalis* DNA, anti-human immunodeficiency virus (HIV) and anti-*Treponema pallidum*, and circulating hepatitis B virus surface antigen (HBsAg) [15] to exclude any systemic and local co-infections in the uterine cervix that could influence the cytokine levels. In addition to serum and ECC samples, we also used samples of cervical biopsies from women positive for HR-HPV with high-grade squamous intraepithelial lesion (HSIL), low-grade squamous intraepithelial lesion (LSIL), and cervical cancer (CC) lesions from previous studies [15, 16].

All women who agreed to participate in this study signed an informed consent form. This study was approved by the Research Ethics Committee of the Federal University of Mato Grosso do Sul and the Fundação Pio XII of Barretos, SP, under protocols n° 1468457 and 1635895, respectively.

### Acquisition of samples

In this study, samples of ECC and serum were used to measure the expression levels of cytokines IFN-γ, TNF-α, IL-10, IL-6, IL-4, and IL-2, and samples of uterine cervix biopsies were used to identify the presence of IL-6 through immunohistochemistry. All samples used in this study were obtained from previous studies [15, 16].

Briefly, the ECC samples for the measurement of cytokines and identification of HPV were collected using disposable specula and soft endocervical brushes with protected tips, as described in a previous study [15]. Serum samples for cytokine measurement were collected via venipuncture. Blood samples were processed to obtain sera, aliquoted, and stored at 80°C until analysis. Samples of uterine cervix biopsies obtained from women positive for HR-HPV DNA through pathological examination of a low-grade squamous intraepithelial lesion (LSIL) (n = 1) and a high-grade squamous intraepithelial lesion (HSIL) (n = 1), and were used for immunohistochemical (IHC) analysis [15]. In addition to these samples, others with pathological classifications such as LSIL (n = 4), HSIL (n = 5), and cervical cancer (n = 4) were included from previous studies [16] to obtain parameters for the evaluation of IL-6 in samples with different degrees of lesion severity.

### Cytokine measurements

The levels of cytokines were determined in the serum and ECCs from 26 patients positive for HR-HPV DNA and 18 patients negative for HPV DNA. The concentrations of IFN-γ, TNF-α, IL-10, IL-6, IL-4, and IL-2 cytokines were determined using a Cytometric Bead Array (CBA) Human Th1/Th2/Th17 Cytokine Kit (BD Biosciences, San Jose, CA, USA). Standards, beads, samples, and protocols for the flow cytometer setup and data acquisition were performed according to the manufacturer’s instructions. Samples were evaluated using a FACS Canto II Flow Cytometer (BD Biosciences, San Jose, CA, USA) and analyzed with the FCAP Array software V. 3.0 (Becton Dickinson, Franklin Lakes, NJ, USA). The results were based on standard concentration curves and expressed as picograms per milliliter (pg/mL).

### Immunohistochemistry

IHC analysis was performed on paraffin-embedded sections to identify the presence of IL-6-positive cells. Immunohistochemical staining was performed following antigen retrieval by pressure cooking for 1 min in 10 mM sodium citrate buffer (pH = 6). IL-6 was detected via incubation with a primary IL-6 antibody, IgG2a (ab9324, Abcam, Cambridge, UK) at a 1:500 dilution for 1 h at room temperature. Detection of immunohistochemical signals was performed using a HiDef Detection Kit (954D-30; Cell Marque, Rocklin, CA, USA) according to the manufacturer’s instructions. Staining was performed with the chromogen 3, 3-diaminobenzidine (EP-12-20542- EasyPath, São Paulo, SP, Brazil) for 10 min. The slides were counterstained with hematoxylin for 20 s. Appropriate positive (with primary antibody) and negative (no primary antibody) controls were included using human tonsil sections.‬‬‬‬‬‬‬‬‬‬‬‬‬‬‬‬‬‬‬‬‬‬‬‬‬‬‬‬‬‬‬‬‬‬‬‬‬‬‬‬‬‬

For sections subjected to immunohistochemistry, images were acquired using a Moticam 2300 digital camera (Motic, Hong Kong, China) attached to a Nikon Eclipse E200 microscope (Nikon, Tokyo, Japan), and were processed using Motic Images Plus 2.0 software (Motic, Hong Kong, China). Images (1024 × 768 pixels) were obtained from 10 randomly selected fields for each slide at 400× magnification without any knowledge of the histopathological lesions. The size of features in the images was determined using ImageJ software 1.47 (National Institutes of Health, Bethesda, MD, USA) and the freehand line tool. Images that were 1024 × 768 pixels corresponded to 170.67 × 128.00 μm. Using the cell counter plugin of ImageJ software, the number of immunostained cells was determined for each image, and the average number of immunostained cells per mm^2^ area was determined [17].

### Statistical analysis

The detection limit values provided in the Cytometric Bead Array (CBA) Human Th1/Th2/Th17 Cytokine Kit (IFN-gamma: 3.7, TNF: 3.8, IL-10: 4.5, IL-6: 2.4, IL-4: 4.9, and IL-2: 2.6) were used for samples whose concentrations were not determined.

The values of cytokine levels are expressed as the median and interquartile range (IQR). Data were analyzed using non-parametric tests. To test the significance of the differences between HR-HPV^+^ and HPV^−^ and single and multiple infections, the Mann-Whitney U-test was used. The Wilcoxon signed-rank test was used to analyze the groups of HR-HPV DNA-positive samples comprising the serum and exfoliated uterine cervix samples from the same patient. The Kruskal-Wallis test was used to compare histopathological and immunohistochemical findings. The results were considered significant at p < 0.05. All statistical analyses were performed using GraphPad Prism 5.0 (GraphPad Software, San Diego, CA, USA).

## Results

The characteristics of the study participants are listed in Table 1. The HR-HPV genotypes identified were HPV 59 (38.5%,10/26), HPV 45 (19.2%, 5/26), HPV 18 (15.4%, 4/26), HPV 16 (11.5%, 3/26), HPV 52 (11.5%, 3/26), HPV 66 (11.5%, 3/26), HPV 33 (3.8%, 1/26), HPV 31 (3.8%,1/26), HPV 26 (3.8%, 1/26), HPV 30 (3.8%, 1/26), HPV 53 (3.8%, 1/26), HPV 69 (3.8%, 1/26), and HPV 82/85 (3.8%, 1/26). The genotypes HPV6/11 (42.3%, 11/26) and HPV32 (3.8%, 1/26) were identified as LR-HPV. Of all the samples, 53.8% (14/26) were classified as single infections caused by only one type of HR-HPV. Multiple infections or infections with more than one viral type were detected in 46.2% (12/26) of the samples [15].

**Table 1:** Characteristics of the study participants (n = 44).

Characteristics	HR-HPV DNA ^+^	HPV DNA^-^
	n (%)	n (%)
**Age**		
< 40 years old	9 (34.6)	8 (44.4)
40–49 years old	7 (26.9)	3 (16.7)
> 50 years old	10 (38.5)	7 (38.9)
**Cytology**		
ASC-US	4 (15.4)	0 (0.0)
NILM	21 (80.8	18 (100.0)
LSIL	1 (3.8)	0 (0.0)

ASC-US, atypical cells of undetermined significance; LSIL, low-grade squamous intraepithelial lesion; HSIL, high-grade squamous intraepithelial lesion.

### Cytokines

The levels of cytokines IFN-γ, TNF-α, IL-10, IL-6, IL-4, and IL-2 were quantified (pg/mL) in the serum and ECCs of 26 patients positive for HR-HPV DNA (mean age: 44.7 years) and 18 patients negative for HPV DNA (mean age: 43.6 years) as shown in the Supporting Information–Cytokine levels (DOI 10.6084/m9.figshare.13550510).

The comparison of cytokine levels in the ECCs and serum of patients positive for HR-HPV revealed a significantly higher concentration of IL-6 in ECCs (p = 0.0135) and IL-10 in the serum (p = 0.0298). The differences in the concentrations of other cytokines were not significant (Table 2). To verify whether HPV influences cytokine secretion, the serum and ECC levels of cytokines were compared between HR-HPV-positive and HPV-negative patients. No significant differences were observed in the blood cytokine levels between the two groups (Table 3). However, IL-6 levels were higher in the ECC samples from HPV-negative patients (p = 0.0132) (Table 3).

**Table 2:** Cytokine levels in the Exfoliated Cervical Cells (ECC) and serum of HR-HPV DNA-positive patients (n = 26).

Cytokines	Serum	ECC	Value of p
	median (25^th^-75^th^)	median (25^th^-75^th^)	
IFN-γ	3.7 (3.7–3.9)	3.7 (3.7–3.7)	0.1952
TNF	4.2 (3.8–4.7)	4.1 (3.8–4.4)	0.4029
IL-10	4.5 (4.5–5.1)	4.5 (4.5–4.5)	0.0247*
IL-6	3.4 (2.4–4.4)	4.6 (2.4–30.2)	0.0135*
IL-4	4.9 (4.9–4.9)	4.9 (4.9–4.9)	0.3125
IL-2	2.6 (2.6–2.6)	2.6 (2.6–2.6)	0.8750

Note: The results are expressed as the median and interquartile range (25^th^-75^th^). Wilcoxon signed-rank test was performed. Statistical significance was set at p < 0.05. ECC exfoliated cervical cells.

**Table 3:** Cytokine levels in the Exfoliated Cervical Cells (ECC) and serum of HR-HPV DNA-positive and HR-HPV DNA-negative patients.

Sample	Cytokines	HPV DNA negative (n = 18)	HR-HPV DNA positive (n = 26)	Value of p
		median (25^th^-75^th^)	median (25^th^-75^th^)	
Serum	IFN-γ	3.7 (3.7–3.7)	3.7 (3.7–3.9)	0.1030
	TNF	3.8 (3.8–4.2)	4.2 (3.8–4.7)	0.0547
	IL-10	4.5 (4.5–4.5)	4.5 (4.5–5.1)	NC
	IL-6	3.6 (2.4–4.5)	3.4 (2.4–4.4)	0.9518
	IL-4	4.9 (4.9–4.9)	4.9 (4.9–4.9)	0.9827
	IL-2	2.9 (2.6–2.6)	2.9 (2.6–2.6)	0.8484
ECC	IFN-γ	3.7 (3.6–3.7)	3.7 (3.7–3.7)	0.3289
	TNF	4.0 (3.9–4.3)	4.1 (3.8–4.4)	0.8658
	IL-10	4.5 (4.5–4.5)	4.5 (4.5–4.5)	0.6842
	IL-6	33.4 (9.4–76.6)	4.6 (2.4–30.2)	0.0132*
	IL-4	4.9 (4.9–4.9)	4.9 (4.9–4.9)	0.8484
	IL-2	2.6 (2.6–2.6)	2.6 (2.6–2.6)	0.1612

Note: The results are expressed as the median and interquartile range (25^th^-75^th^). The Mann-Whitney U-test was performed, and statistical significance was set at p < 0.05. ECC, exfoliated cervical cells. NC, not calculated.

To investigate whether a simple infection (by a single viral genotype) or multiple infections (by more than one viral genotype) influences the cytokine profile in the serum and ECCs, the cytokine levels were compared between both sites in these two groups (patients with simple infection and multiple infections). IL-6 levels were higher in the ECCs of patients with multiple infections (p = 0.0380) than those patients with a simple infection (Table 4), although the levels were lower than those detected in patients negative for HPV DNA (Table 3). The concentrations of other cytokines were similar between the two groups. Evaluation of serum samples showed no significant differences in the concentrations of cytokines between the different groups based on the infection type (Table 4).

**Table 4:** Cytokine levels in the Exfoliated Cervical Cell (ECC) and serum of patients with single and multiple infections.

Sample	Cytokines	Simple Infection (n = 14)	Multiple Infection (n = 12)	Value of p
		median (25^th^-75^th^)	median (25^th^-75^th^)	
Serum	IFN-γ	3.7 (3.7–4.8)	3.7 (3.7–3.7)	0.4646
	TNF	3.9 (3.8–4.3)	4.5 (3.9–5.2)	0.1180
	IL-10	4.5 (4.5–5.1)	4.5 (4.5–5.0)	0.7922
	IL-6	3.3 (2.4–4.6)	3.4 (2.7–4.4)	0.6966
	IL-4	4.9 (4.9–4.9)	4.9 (4.9–4.9)	0.6103
	IL-2	2.6 (2.6–2.6)	2.6 (2.6–2.6)	NC
ECC	IFN-γ	3.7 (3.7–3.8)	3.7 (3.7–3.9)	0.8891
	TNF	4.1 (3.8–4.4)	4.1 (3.8–5.3)	0.6381
	IL-10	4.5 (4.5–4.5)	4.5 (4.5–4.5)	NC
	IL-6	3.0 (2.4–8.3)	16.10 (2.8–52.7)	0.0380*
	IL-4	4.9 (4.9–4.9)	4.9 (4.9–4.9)	0.9114
	IL-2	2.6 (2.6–2.6)	2.6 (2.6–2.6)	NC

Note: The results are expressed as the median (25^th^-75^th^ percentile). The Mann-Whitney U-test was performed, and statistical significance was set at p <0.05. * p < 0.05. ECC, exfoliated cervical cells. NC, not calculated.

In the analysis of cytokine levels in serum and ECC samples between different age groups, we found that IL-6 was significantly higher in serum samples from positive HR-HPV DNA patients older than 50 years (p = 0.0195) than in the group under 40 years old (median and IQR for IL-6: 4.4, 3.45. 5.6; 3.4, 2.5. 3.9, respectively).

### Cells expressing IL-6 in uterine cervix lesions

To confirm the role of IL-6 in the cervical microenvironment of patients infected with HR-HPV, samples with different degrees of cervical lesions were subjected to immunohistochemistry analysis. Immunostaining for IL-6 was performed on 15 samples from HR-HPV-positive uterine cervix biopsies, two of which were obtained from the patients at the Hospital de Amor de Campo Grande (Barretos Cancer Hospital), Campo Grande, MS, and 13 were derived from a previous study [16]. Among the 15 samples, 5, 6, and 4 were classified as LSIL, HSIL, and cervical cancer, respectively.

**Fig 1:**
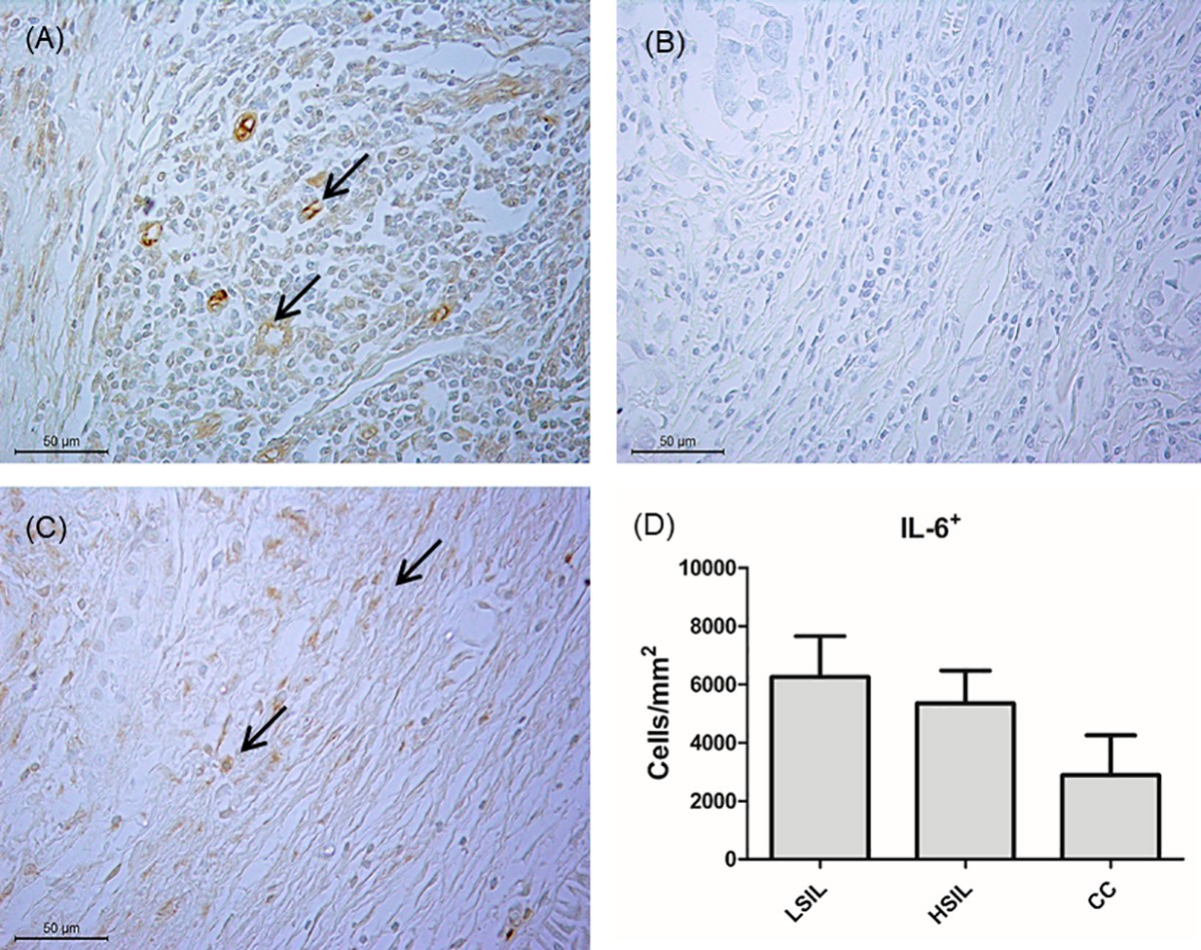
Cells expressing IL-6. (A) Human tonsil sections labeled with primary anti-human IL-6 antibody. (B) Human tonsil sections without primary anti-human IL-6 antibody. (C) Uterine cervix biopsies labeled with primary anti-human IL-6 antibody. The black arrow corresponds to IL-6^+^ cells. All figures are presented in the same magnification (400×). (D) The number of cells expressing IL-6 in samples of uterine-cervix biopsies with histopathological lesions.

In total, 150 images were acquired and analyzed to count cells expressing IL-6 in the stromal samples of uterine cervix biopsies (Fig 1). The mean number of IL-6^+^ cells varied from 2883.8 to 6252.9 cells/mm^2^, but there was no significant difference among the histopathological classes (Fig 1).

## Discussion

The results of this study demonstrate the differences in the levels of IL-6 and IL-10 among patients positive for HR-HPV, as well as the difference in their immune response at the local and systemic levels. IL-6 levels were elevated in the ECCs from HPV-negative patients, suggestive of its continuous stimulation at this site owing to the existing microbiota with pro-inflammatory activities [18]. Furthermore, IL-6 concentration in the ECCs of HR-HPV-positive patients and those with multiple infections was high but lower than that reported in HPV-negative patients. IL-10 expression may be responsible for the suppression of various cytokines, including IL-6 [19]. The level of this immunosuppressive cytokine was systemically high in HR-HPV-positive patients. IHC analysis for the expression level of IL-6 *in situ* revealed no differences between samples with different degrees of histopathological lesions.

Persistent HR-HPV infection is an important factor in the development of cervical cancer [2, 3, 20]. Although HPV16 and HPV18 are the genotypes most closely related to cervical cancer, other genotypes have also been associated with cancer and its precursor lesions and may vary according to age and geographic region [3, 20, 21].

Global epidemiological studies have indicated 18 different oncogenic high-risk HPV genotypes (HPV16, HPV18, HPV26, HPV31, HPV33, HPV35, HPV39, HPV45, HPV51, HPV52, HPV53, HPV56, HPV58, HPV59, HPV66, HPV68, HPV73, and HPV82) that are associated with cervical cancer [21, 22]. HPV69, HPV30, and HPV85 were regarded as highly oncogenic in the present study, although they were classified as possibly oncogenic [23].

Since the immune response is essential for the elimination of HPV, and age and hormonal changes could influence the immune system and affect the mechanisms of viral elimination [24], cytokine level analysis may be useful to better understand the changes and deviations in the immune response caused by HPV infection at the systemic and local levels. In our study, we found that the concentration of IL-6 in serum samples from HR-HPV-positive patients was higher in the group of women over the age of 50, when compared to those under the age of 40 (p = 0.0195), suggesting that in this group, there may be a greater stimulus for the production of this cytokine at the systemic level. However, additional studies are needed to assess whether this increase may be due to persistent HR-HPV infection or whether it is associated with possible hormonal variations and other aging stimuli.

Several studies have investigated the relationship between cervical cancer and its precursor lesions and the levels of cytokines present in the serum, cervical secretions, and uterine cervix biopsies [11, 13, 25, 26]. However, the expression of cytokines analyzed in the serum and ECC samples came mostly from patients without cervical lesions, which allowed us to evaluate the relationship between the host immune system and the virus.

The comparison of IL-6 levels in the serum and ECCs of HR-HPV-positive patients revealed higher levels of IL-6 in the ECCs, suggesting that IL-6 is mainly produced at the site of infection and that the immune response in the same patient changes at the systemic level. However, the level of IL-6 was 8.3-fold higher in the ECCs of HPV-negative patients. In a study evaluating IL-6 concentration in the cervicovaginal washings of patients with cervical cancer and cervical intraepithelial neoplasia (CIN) and healthy controls, higher IL-6 levels were observed in patients with cervical cancer than in healthy subjects, indicating a relationship between IL-6 levels and the pathogenesis and severity of cervical neoplasia [13]. Unlike in the present study, these samples were obtained from HPV-positive patients diagnosed with cervical cancer or CIN. This may explain the higher concentration of IL-6 at this site [27], as stromal cells or neoplastic cells tend to release more IL-6, IL-8, and vascular endothelial growth factor (VEGF) at sites of intense inflammation (chronic infection and cervical lesions) [27–29]. Zanotta *et al*. [11] evaluated cytokine levels in the cervicovaginal fluid of young patients with and without cervical lesions infected with HR-HPV and observed an association between IL-6 expression and HR-HPV infection. These authors also reported a significant increase in IL-6 expression in samples with cytological abnormalities compared with HR-HPV-positive and -negative samples without cytological abnormalities. Thus, HR-HPV may influence IL-6 production during the early inflammatory response.

The expression of IL-6 was observed to be higher in ECCs than in the serum. IL-6 is capable of promoting the growth of cervical tumor cells [30] and is involved in tumor angiogenesis through the regulation of VEGF during the early stages of cervical cancer [27, 31]. Furthermore, IL-6 is associated with the development of HPV-induced cervical lesions [13, 27]. Hence, IHC analysis was performed to detect the expression of IL-6 in the stroma from samples with different degrees of cervical lesions. However, no relevant changes were observed. Other authors that assessed the levels of IL-6 in cervical cancer tissues and adjacent non-tumor tissues found higher expression in cervical cancer tissues [27, 32], suggesting its role as a biomarker for the prognosis and therapy of cervical cancer [32].

As IL-6 may induce the differentiation of activated B cells into antibody-producing cells [33] and CD8^+^ T cells into cytotoxic cell types [34], its presence in the cervix of healthy individuals is of great importance for the elimination and control of various microorganisms. The presence of IL-6 at high concentrations in the exfoliated uterine cervix of HPV-negative patients, as observed in the present study, may be due to the constant stimuli at this site from the naturally colonizing microorganisms [35, 36]. IL-6 concentration at this site remained high in HR-HPV-positive patients but was lower in HPV-negative patients. This observation may be related to the immunosuppressive stimuli produced by HPV, such as the induction of IL-10 production by different cell types in the infected microenvironment [17, 37] or even by systemic IL-10 [38]. This study revealed a higher concentration of IL-10 in the serum than in the cervix of HPV-positive patients. In the serum, IL-10 may play a role in controlling IL-6 production and subsequently decreasing its concentration in the blood. However, the suppression triggered by systemic IL-10 was insufficient to completely inhibit the production of IL-6 in the cervix.

IL-10 is capable of modulating both innate and adaptive immunity, primarily because it exerts anti-inflammatory effects, and is therefore essential for maintaining homeostasis of the immune response [39]. Upon overproduction, IL-10 may impair the immune response by inhibiting the production of IL-2, IFN-γ, and IL-12, which are essential for virus and tumor cell clearance. Some authors have associated the presence of high levels of IL-10 with persistent HPV infection and the progression of cervical lesions induced by this virus [12, 40]. However, in the present study, no association was observed between cytokine levels and the presence or absence of HPV infection. In HR-HPV-positive patients, a difference in cytokine levels was noted at the site of analysis (local or systemic). Thus, HPV infection, although considered restricted to the epithelium, could induce the systemic production of IL-10.

Studies have shown that cervical infections caused by more than one type of HPV are frequently reported [41, 42], as infections by a specific virus type do not reduce the likelihood of infection by other phylogenetically related types [43]. In the present study, 12 patients with multiple infections were detected, and their cytokine profiles were analyzed at the systemic and local levels and compared with those of the patients with simple infection. IL-6 expression was significantly higher in the ECCs of patients with multiple infections. In these patients, simultaneous infection with several viral genotypes may induce a more vigorous local immune response compared to patients infected by a single viral genotype, consistent with the increase in IL-6 expression level in the ECCs. Unfortunately, in our study, it was not possible to form a group of patients with multiple infections caused only by HR-HPV, excluding any other type of LR-HPV that may interfere with these results.

The data of this study on IL-6 and the data published [15] about IL-17 in the same population show that HPV plays an important role in immune response modulation. The induction of Th17 responses producing IL-17 causes viral persistence and difficulty in the removal of infected cells by inhibiting cytotoxic T cell function and apoptotic cell death. IL-6 is a major cytokine that promotes the induction of pathogenic Th17 responses [44]. The effects of both cytokines IL-6 and IL-17 have a major impact on several chronic diseases, such as chronic viral infection and cancer development. An elevation in the expression of these cytokines can play a critical role in establishing viral persistence and the development of cervical cancer.

In conclusion, this study provides evidence that IL-6 expression was significantly increased in ECCs (p = 0.0135) during HPV infection. Regarding IL-10, although the difference in concentration between the two sites (ECC and serum) was not very high, it was significant, showing that there were differences in the expression of this cytokine at the systemic and local levels. This finding, in addition to the fact that IL-6 and IL-10 are related to the development of HPV-induced cervical lesions and suppression of the immune cells that are important in virus elimination, suggests that these patients are at a higher risk of persistent HPV infection. Further studies are warranted to monitor the development of infections in these patients.

## Supporting information

S1 Table Primes used in pool PGMY-PCR detection according to fragment size, sequence, annealing region and melting temperature (Tm). (DOCX)

S2 Table Primes used in TS-PCR genotyping according to fragment size, sequence, annealing region and melting temperature (Tm). (DOCX)
