# Supplementary material for: IL-6 and IL-10 in the serum and exfoliated cervical cells of patients infected with high-risk human papillomavirus
Source: PLoS One. 2021 Mar 22;16(3):e0248639. doi: 10.1371/journal.pone.0248639 (PMC7984643; doi:10.1371/journal.pone.0248639)
Supplement: S1 Table — (DOCX) [file pone.0248639.s001.docx]

**Primers**

**S1 Table- *Primes used in pool PGMY-PCR detection according to fragment size, sequence, annealing region and melting temperature (Tm).***

| ***Primer***  **(name)** | **Sequence**  **(5’-3’)** |
| --- | --- |
| PGMY -11A | GCACAGGGACATAACAATGG |
| PGMY -11B | GCGCAGGGCCACAATAATGG |
| PGMY -11C | GCACAGGGACATAATAATGG |
| PGMY -11D | GCCCAGGGCCACAACAATGG |
| PGMY -11E | GCTCAGGGTTTAAACAATGG |
| PGMY -O9F | CGTCCCAAAGGAAACTGATC |
| PGMY -O9G | CGACCTAAAGGAAACTGATC |
| PGMY -O9H | CGTCCAAAAGGAAACTGATC |
| PGMY -O9I | GCCAAGGGGAAACTGATC |
| PGMY -O9J | CGTCCCAAAGGATACTGATC |
| PGMY -O9K | CGTCCAAGGGGATACTGATC |
| PGMY -O9L | CGACCTAAAGGGAATTGATC |
| PGMY -O9M | CGACCTAGTGGAAATTGATC |
| PGMY -O9N | CGACCAAGGGGATATTGATC |
| PGMY -O9P | GCCCAACGGAAACTGATC |
| PGMY -O9Q | CGACCCAAGGGAAACTGGTC |
| PGMY -O9R | CGTCCTAAAGGAAACTGGTC |
| HMBO1 | GCGACCCAATGCAAATTGGT |

Note: GRAVITT, P. E.; PEYTON, C. I.; ALESSI, T. Q.; WHEELER, C. M.; COUTLÉE, F.; HILDSHEIM, A.; SCHIFFMAN, M. H.; SCOTT, D. R.; APPLE, R. J. Improved amplification of genital human Papillomaviruses. Journal Clinical Microbiology, v. 38, n. 1, p. 357-361, Jan. 2000.
