# Supplementary material for: IL-6 and IL-10 in the serum and exfoliated cervical cells of patients infected with high-risk human papillomavirus
Source: PLoS One. 2021 Mar 22;16(3):e0248639. doi: 10.1371/journal.pone.0248639 (PMC7984643; doi:10.1371/journal.pone.0248639)
Supplement: S2 Table — (DOCX) [file pone.0248639.s002.docx]

**Primers**

**S2 Table. Primes used in TS-PCR genotyping according to fragment size, sequence, annealing region and melting temperature (Tm).**

| ***Primer***  **(F-R)** | **Fragment**  **(pb)** | **Sequence**  **(5’-3’)** | **Region** | **Tm (°C)** |
| --- | --- | --- | --- | --- |
| HPV 6 F^(1)^ | 194 | CACGTCTGCAACGACCATAG | E6 | 51,4 |
| HPV 6 R^(1)^ |  | CCATGAAATTCTAGGCAGCA | E6 | 50,4 |
| HPV 11 F^(2)^ | 210 | TCTGTGTCTAAATCTGATAC | L1 | 38,8 |
| HPV 11 R^(2)^ |  | GGGTTTCTGACAGGTAATGGC | L1 | 52,6 |
| HPV 16 F^(3)^ | 174 | TTGCAGATCATCAAGAACACGTAGA | E6-E7 | 59,8 |
| HPV 16 R^(3)^ |  | CTTGTCCAGCTGGACCATCTATTT | E6-E7 | 59,5 |
| HPV 18 F^(3)^ | 219 | CAACCGAGCACGACAGGAA | E6-E7 | 59,4 |
| HPV 18 R^(3)^ |  | CTCGTCGGGCTGGTAAATGTT | E6-E7 | 60,0 |
| HPV 31 F^(4)^ | 420 | GATGCAACGTGCTCAGGG | L1 | 54,2 |
| HPV 31R^(4)^ |  | GCGACCCAGTGGAAACTGATCTA | L1 | 57 |
| HPV 33 F^(3)^ | 90 | AAACCTTTGCAACGATCTGAGGTA | E6 | 60,0 |
| HPV 33 R^(3)^ |  | GTTTACATATTCCAAATGGATTTCCCTCTCT | E6 | 63,4 |
| HPV 45 F^(5)^ | 195 | ACCAGATTTGTGCACAGAAT | E6/E7 | 46,7 |
| HPV 45 R^(5)^ |  | TTTTTTCCAGTGTCTCTCCA | E6/E7 | 47,1 |

Note: ^(1)^SILVA *et al.,* 2003; ^(2)^LIN *et al.,* 2008; ^(3)^GUO *et al.,* 2007; ^(4)^SWAN *et al.,* 1999; ^(5)^KARLSEN *et al.,* 1996.

**References**

GUO, M.; SNEIGE, N.; SILVA, E. G.; JAN, Y. J.; COGDELL, D. E.; LIN, E.; LUTHRA, R.; ZHANG, W. Distribution and viral load of eight oncogênico types of human papillomavirus (HPV) and HPV 16 integration status in cervical intraepithelial neoplasia and carcinoma.**Modern Pathology**, v. 20, n. 2, p. 256-266, Feb. 2007.

KARLSEN, F.; KALANTARI, M.; JENKINS, A.; PETTERSEN, E.; KRISTENSEN, G.; HOLM, E.;JOHANSSON, B.; , B.; Use of multiple PCR primer sets for optimal detection of human Papillomavirus. **Journal of Clinic Microbiology**, v. 34, n. 9, p. 2095-2100, Sept. 1996.

LIN, C. Y., CHAO, A., YANG, Y.C., CHOU, H. H., HO, C. M., LIN, R. W., CHANG, T. C., CHIOU, J. Y., CHAO, F. Y., WANG, K. L., CHIEN, T. Y., HSUEH, S., HUANG, C. C., CHEN, C. J., LA, I. C.H., Human papillomavirus typing with a polymerase chain reaction-based genotyping array compared with type-specific PCR.**JournalClinicalVirology,** v. 42, n.2, p. 361-367, Jan. 2008.

SILVA, A. M. T. C., O Papel do Papiloma Vírus Humano na Carcinogênese. **Revista Biotecnologia**, v.29, p.48-54, Abr. 2003.

SWAN, D. C.; TUCKER, R. A.; TORTOLERO-LUNA, G.; MITCHELL, M. F.; WIDEROFF, L.; UNGER, E. R. Human papillomavirus (HPV) DNA copy number is dependent on grade of cervical disease and HPV Type. **Journal Clinical Microbiology**, v. 37, n. 4, p. 1030-1034, 1999.
